# Supplementary material for: A light-regulated host–guest-based nanochannel system inspired by channelrhodopsins protein
Source: Nat Commun. 2017 Aug 15;8:260. doi: 10.1038/s41467-017-00330-z (PMC5558008; doi:10.1038/s41467-017-00330-z)
Supplement: Supplementary file 1 — Supplementary Information [file 41467_2017_330_MOESM1_ESM.pdf]

File Name: Supplementary Information
Description: Supplementary Figures, Supplementary Methods and Supplementary References

File Name: Peer Review File
Description:

Supplementary Figures

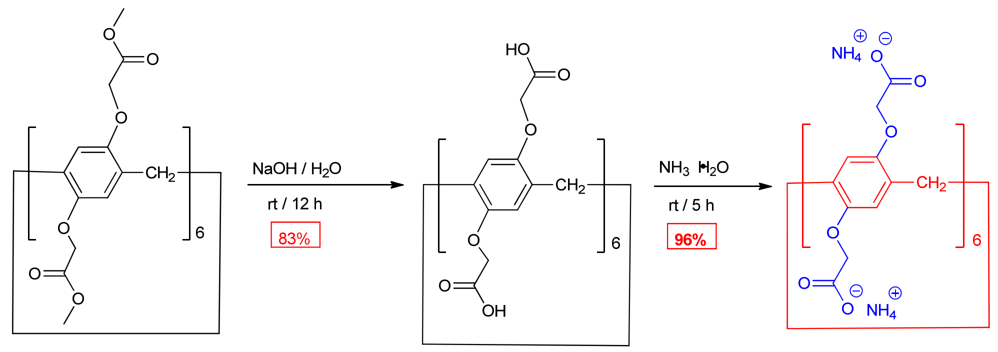

Supplementary Figure 1. The synthesis route to negative host P6A. Synthesis of compound P6A: Compound P6A was synthesized according to the literature.[1]

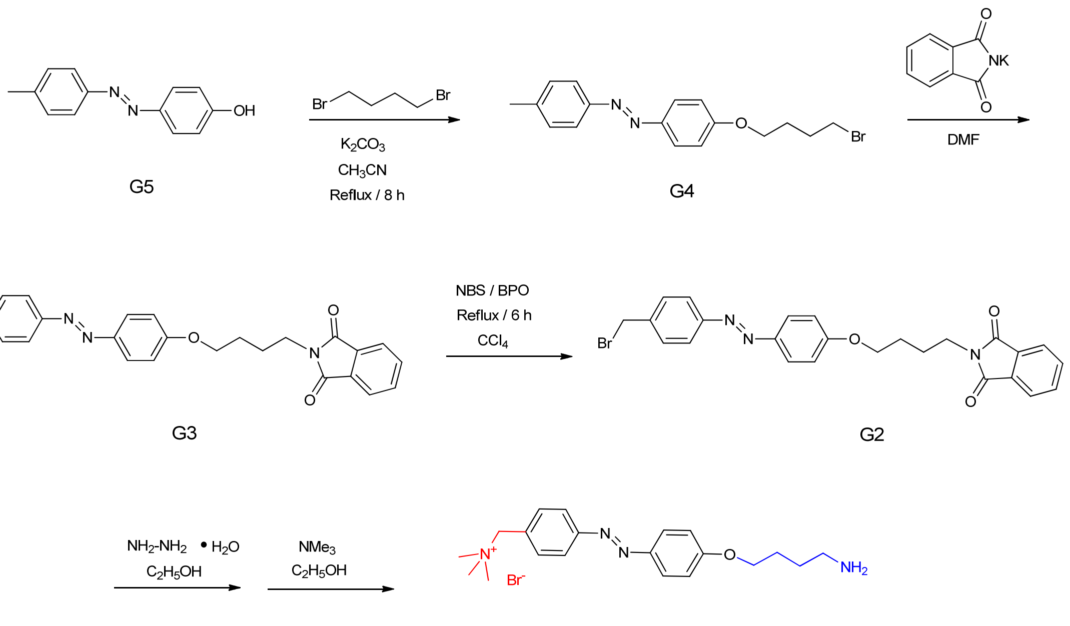

Supplementary Figure 2. The synthesis route for positive AZO guest.

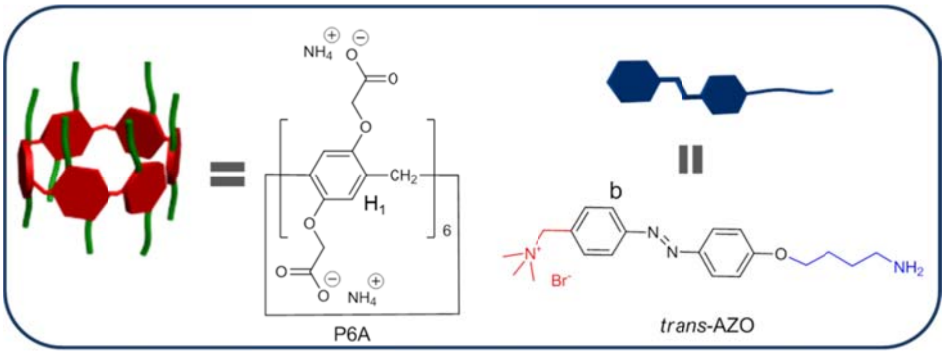
P6A
H1
6
trans-AZO
b

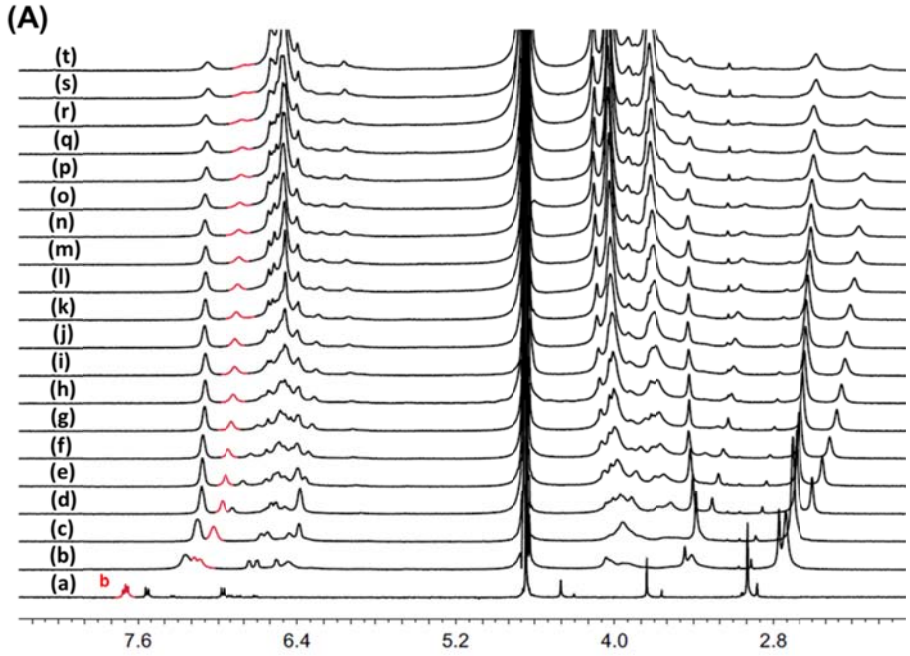
(A)
(t)
(s)
(r)
(q)
(p)
(o)
(n)
(m)
(l)
(k)
(j)
(i)
(h)
(g)
(f)
(e)
(d)
(c)
(b)
(a)
b
7.6
6.4
5.2
4.0
2.8

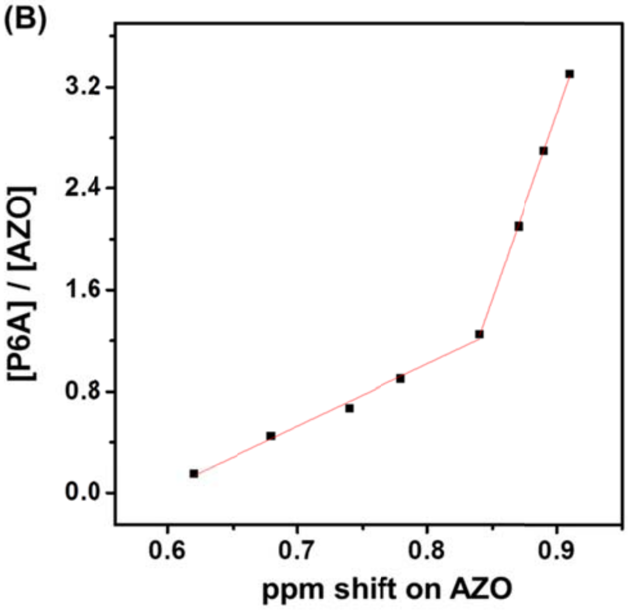
(B)
[P6A] / [AZO]
3.2
2.4
1.6
0.8
0.0
0.6
0.7
0.8
0.9
ppm shift on AZO

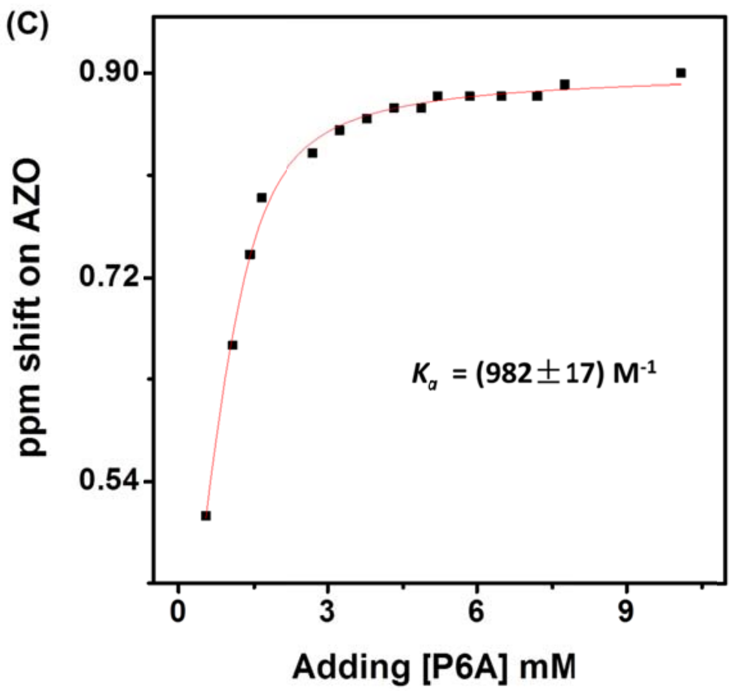

Supplementary Figure 3. The interaction between AZO and P6A. (A) ^{1}H NMR (600 MHz) binding study of complex P6A vs AZO in D_2O; (B) The mole ratio plot for the complexation between P6A and AZO, indicating a 1:1 stoichiometry; (C) The non-linear curve-fitting (NMR titrations) for the complexation of AZO (4.0 mM) with different concentration of P6A. The association constant (K_a) of P6A and AZO was calculated to be about 982 M^{-1}.

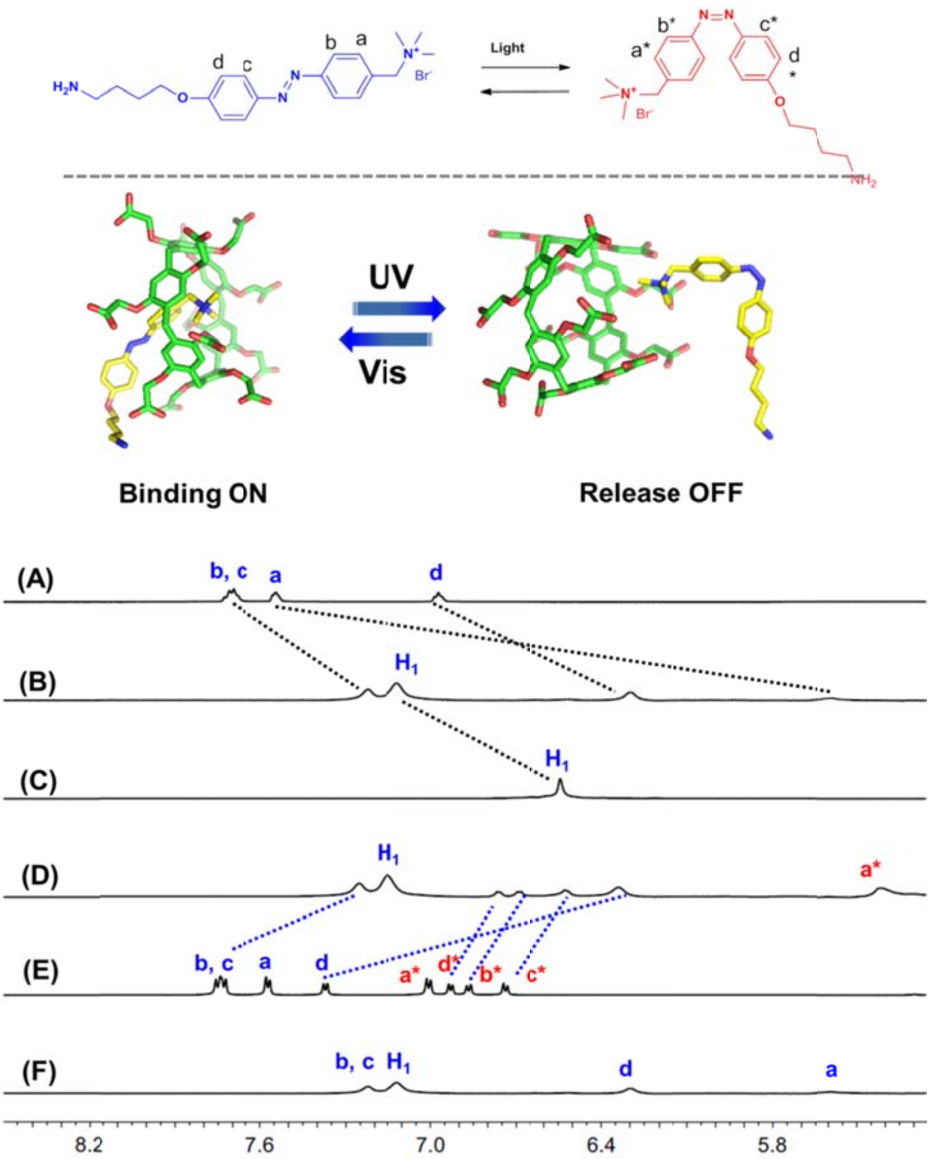

Supplementary Figure 4. The photocontrollable threading–dethreading behavior between P6A and AZO. Partial ^{1}H NMR spectra (400 MHz, D_2O, room temperature): (A) *trans*-AZO (3.0 mM); (B) *trans*-AZO (3.0 mM) and P6A (3.0mM); (C) P6A (3.0 mM); (D) *trans*-AZO (3.0 mM) and P6A (3.0 mM) after irradiation at 365 nm for 15 min; (E) *trans*-AZO (3.0 mM) after irradiation at 365 nm for 15 min; (F) *trans*-AZO (3.0 mM) and P6A (3.0 mM) after further irradiation at 435 nm for 15 min.

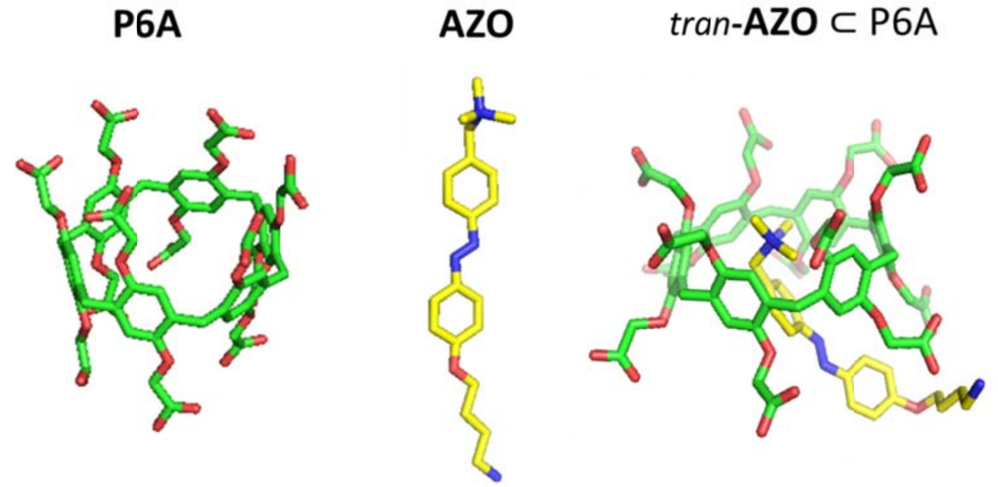

Supplementary Figure 5. Molecular stimulation. Energy-minimized complex of P6A with AZO, optimized at the B3LYP/6–31G* level.

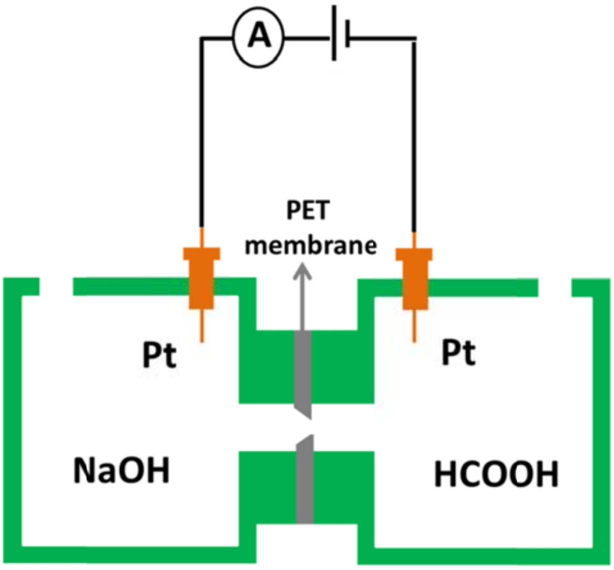

Supplementary Figure 6. Fabrication of single conical nanochannel. Schematic image for etching conical nanochannel in a conductivity cell.

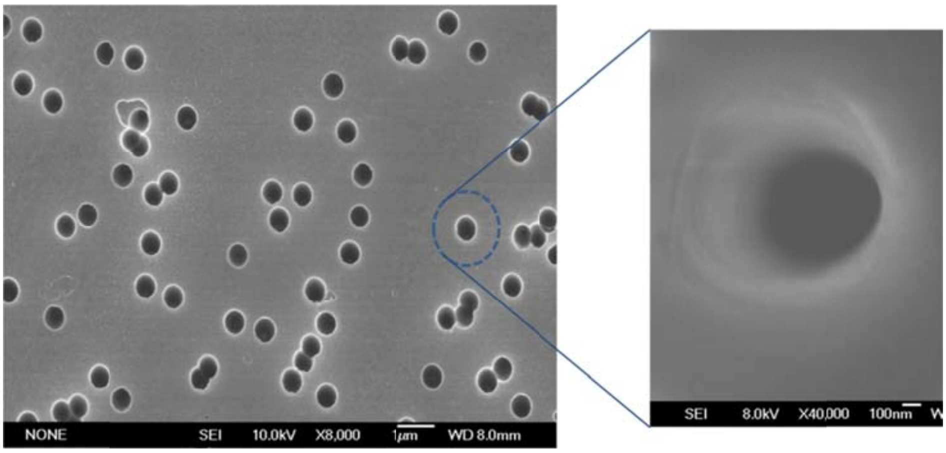

Supplementary Figure 7. SEM characterization of nanochannel. SEM image of the base side and the tip side of the conical nanochannel in PET porous membrane channels (10^7channels cm^{-2}). It shows that the diameter of the large opening (base) of the conical nanochannel was approximately 600 nm, while that of the narrow opening (tip) at the opposite face was approximately 20 nm.

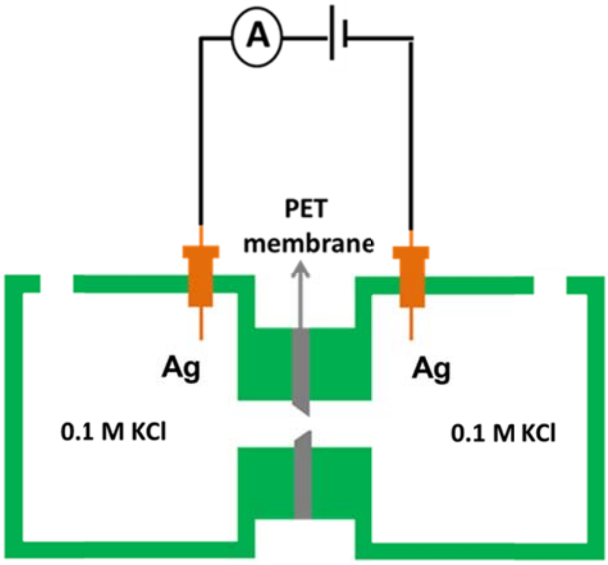

Supplementary Figure 8. Ion currents measurement. The experiments of measuring the resulting ion current flowing through the nanochannel.

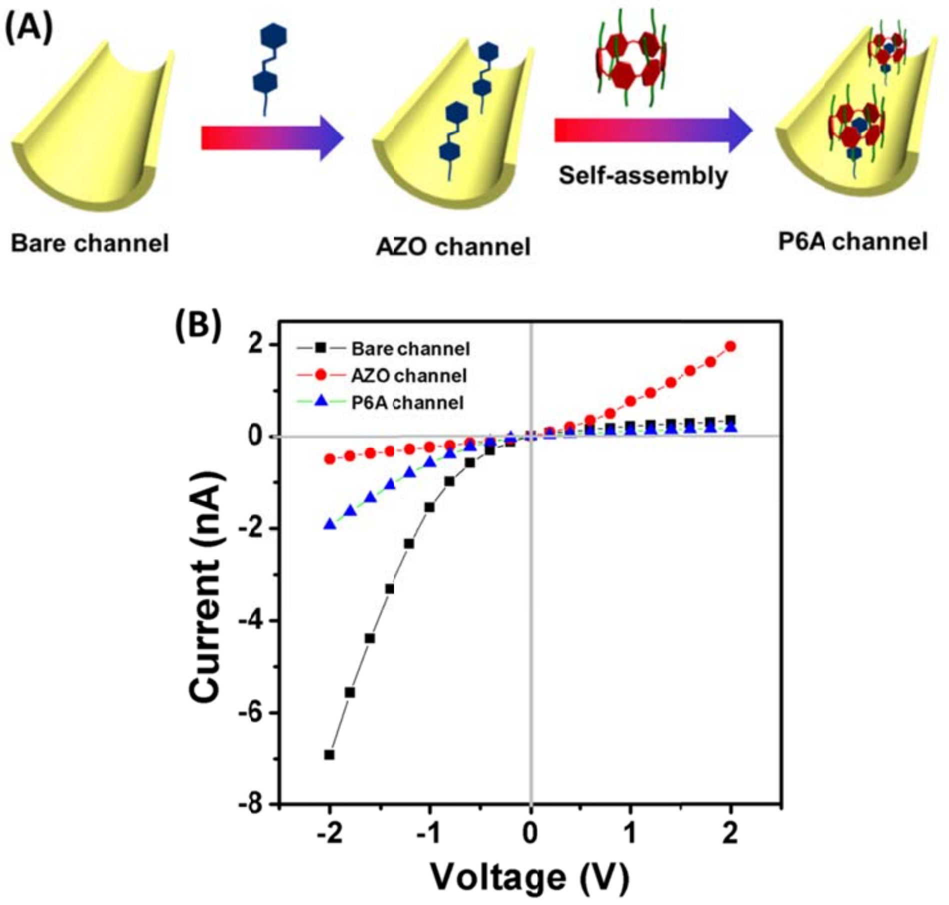

Supplementary Figure 9. The modification of light-controlled nanochannel. (A) Schematic description of modification process in nanochannel; (B) *I–V* characteristics of P6A-assembled nanochannel. The result shows that the P6A was coupled to the inner surface of the nanochannel successfully.

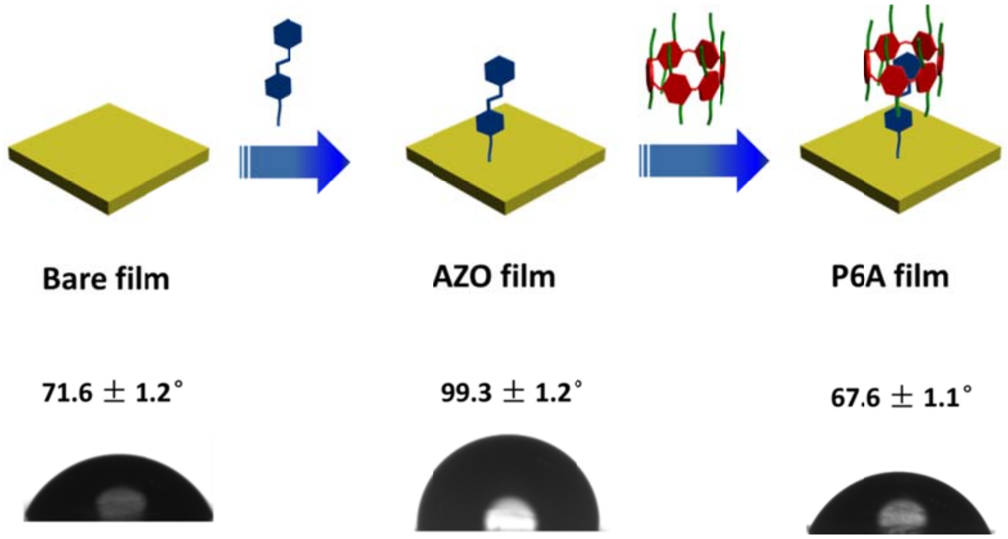

Supplementary Figure 10. Contact angles measurement. The wettability change of P6A-assembled nanochannel. The result shows that light-activated nanochannel was coupled to the inner surface of the nanochannel successfully.

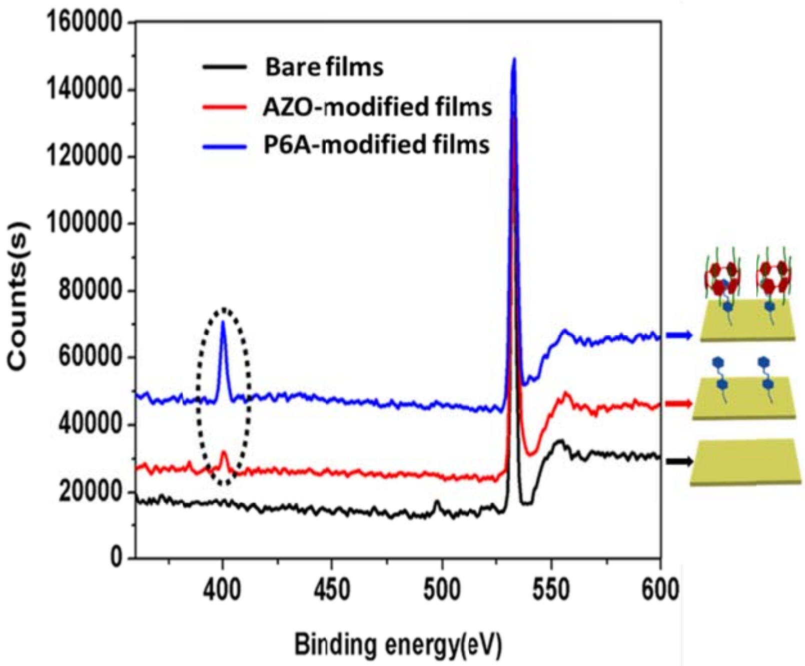

Supplementary Figure 11. XPS experiment. XPS spectra of PET films before and after modification. The control was referenced to the bare film (black). The modified AZO was referenced to the film after the modification of AZO (red), and the modified P6A was referenced to the film after the modification of P6A (blue). The results indicate that light-controlled was modified on the surface of the film successfully.

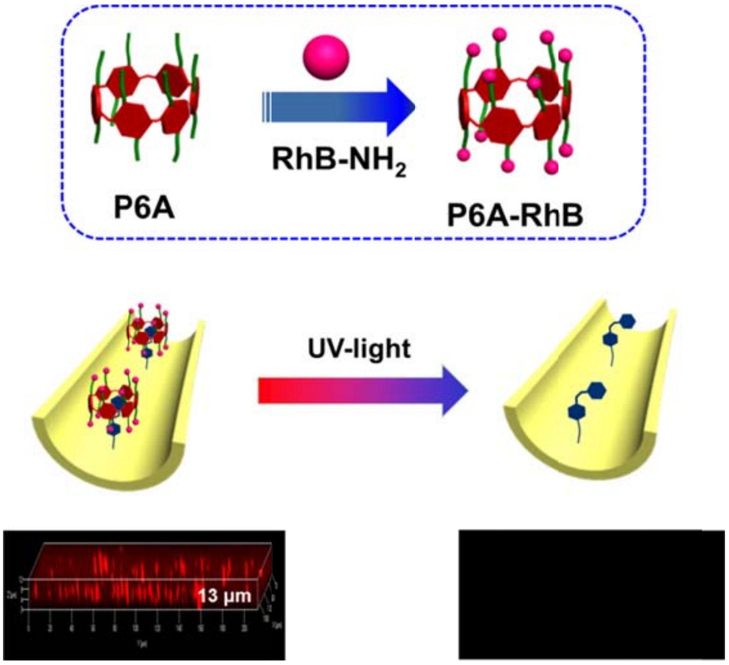

Supplementary Figure 12. Laser scanning confocal microscopy experiment. Laser scanning confocal microscopy (LSCM) images observed the fluorescence change of the nanochannel towards UV light irradiation

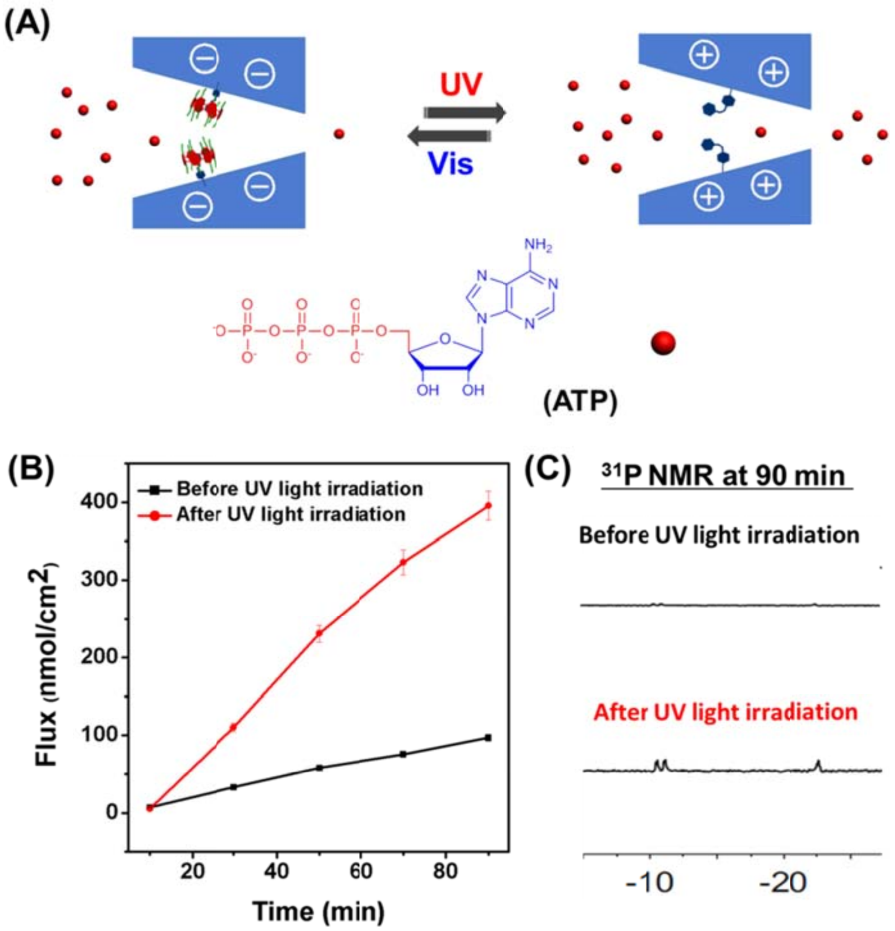

Supplementary Figure 13. Molecules transport of the ATP. (A) Schematic illustration of the transport of molecules through the multi-channel membrane before and after light irradiation; (B) Permeation data for ATP before and after UV light irradiation of the AZO-P6A-modified channel; (C) ^{31}P NMR at 90 min transport before and after UV light irradiation.

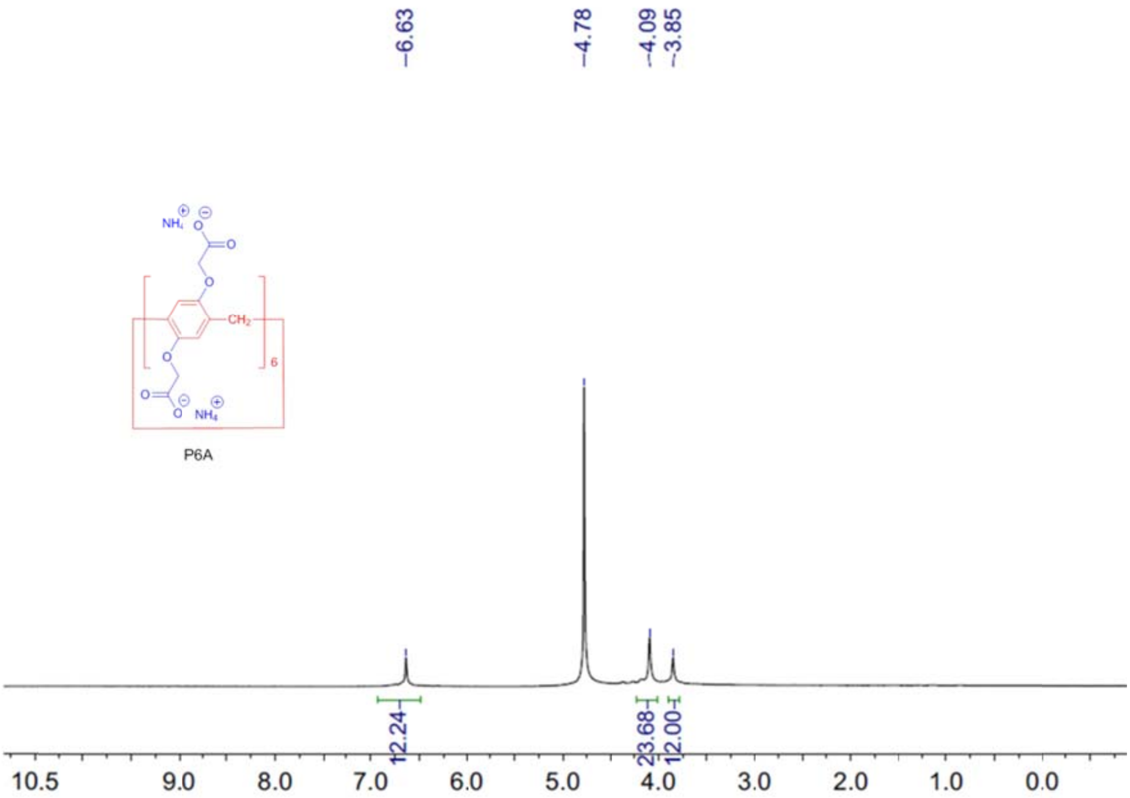

Supplementary Figure 14. ^{1}H NMR spectrum (400 MHz) of P6A in D_2O.

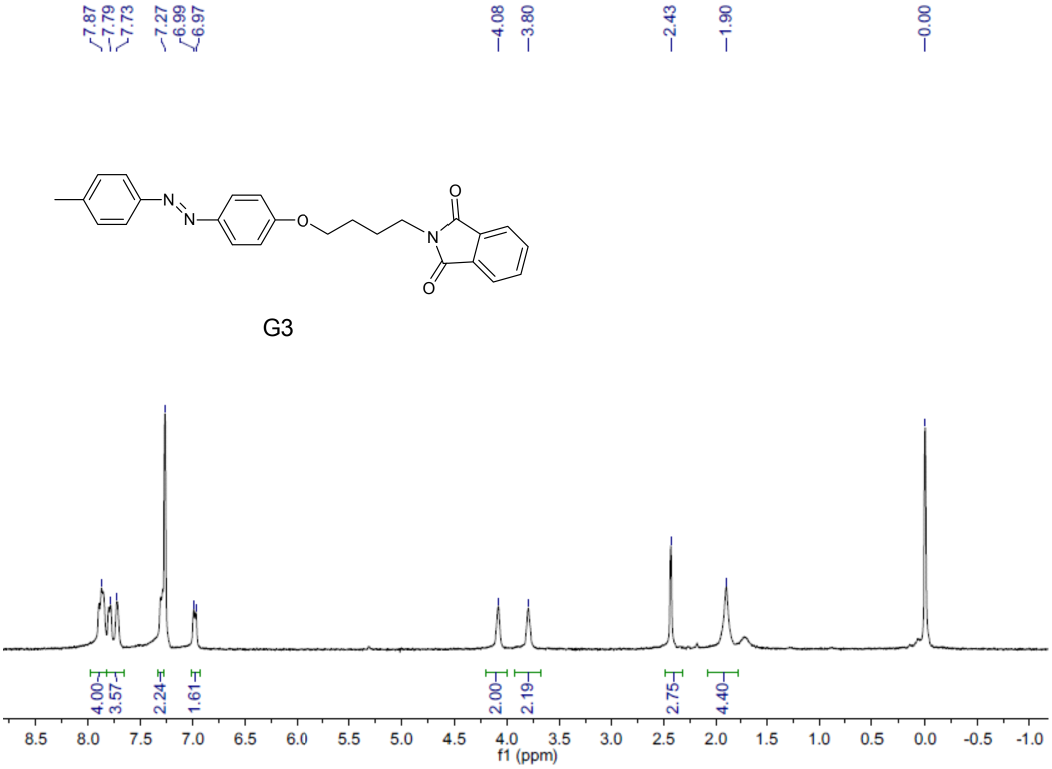

Supplementary Figure 15. ^{1}H NMR spectrum (400 MHz) of compound **G3** in $CDCl_3$.

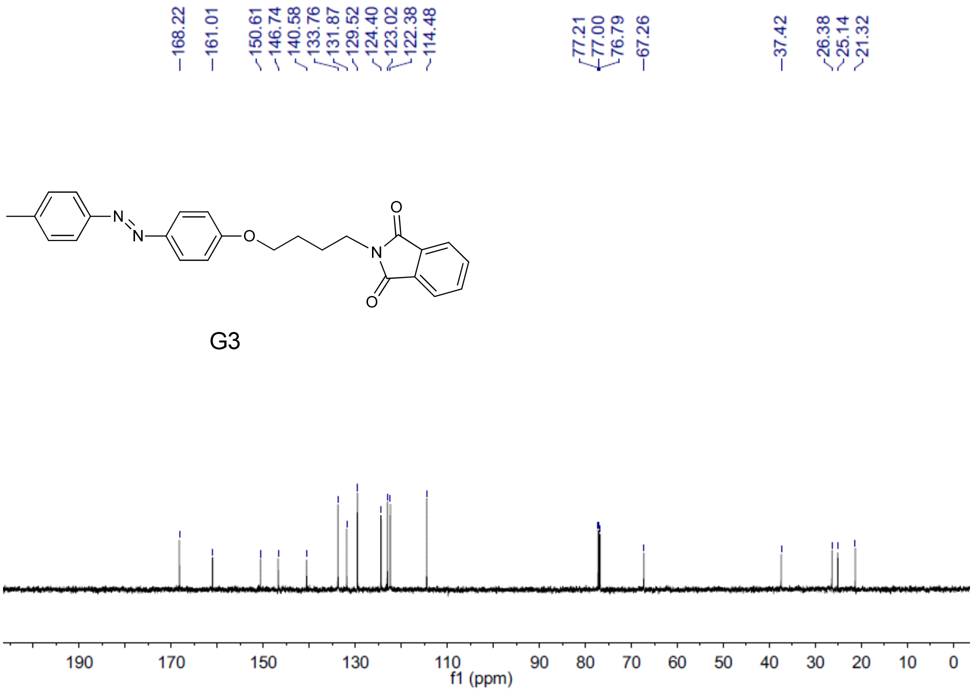

Supplementary Figure 16. ^{13}C NMR spectrum (100 MHz) of **G3** in $CDCl_3$.

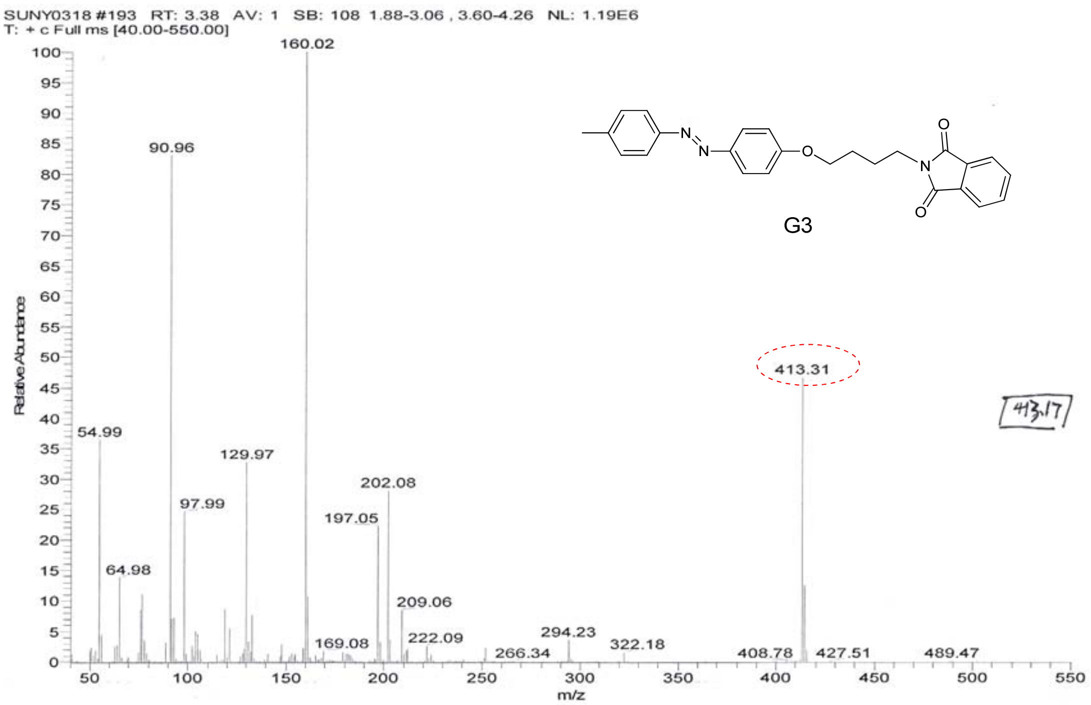

Supplementary Figure 17. Mass spectrum of compound **G3**.

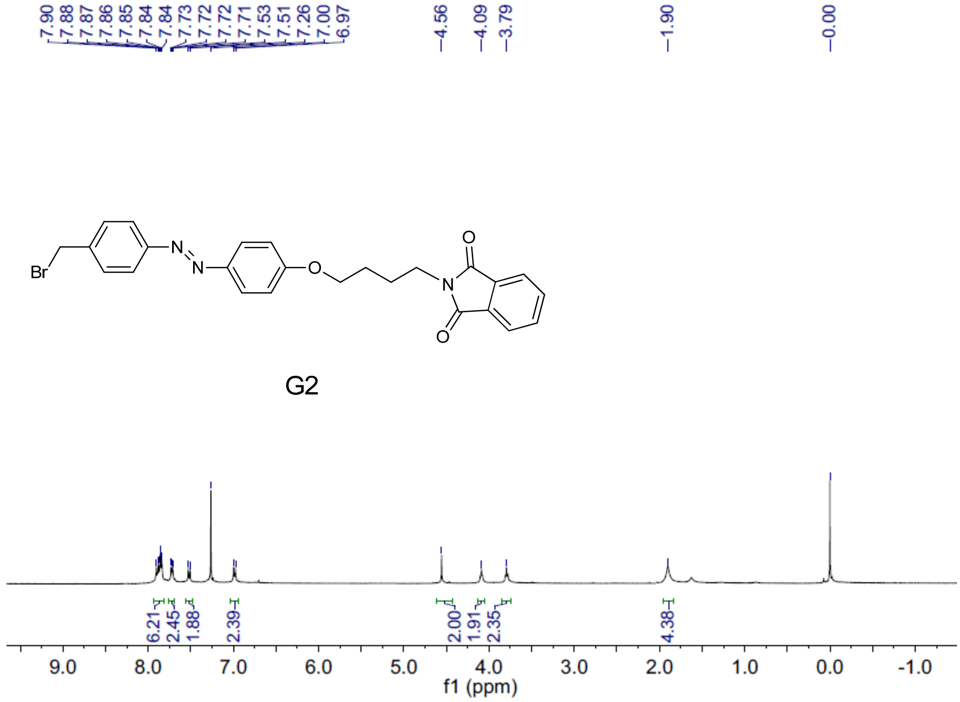

Supplementary Figure 18. ^{1}H NMR spectrum (400 MHz) of **G2** in $CDCl_3$.

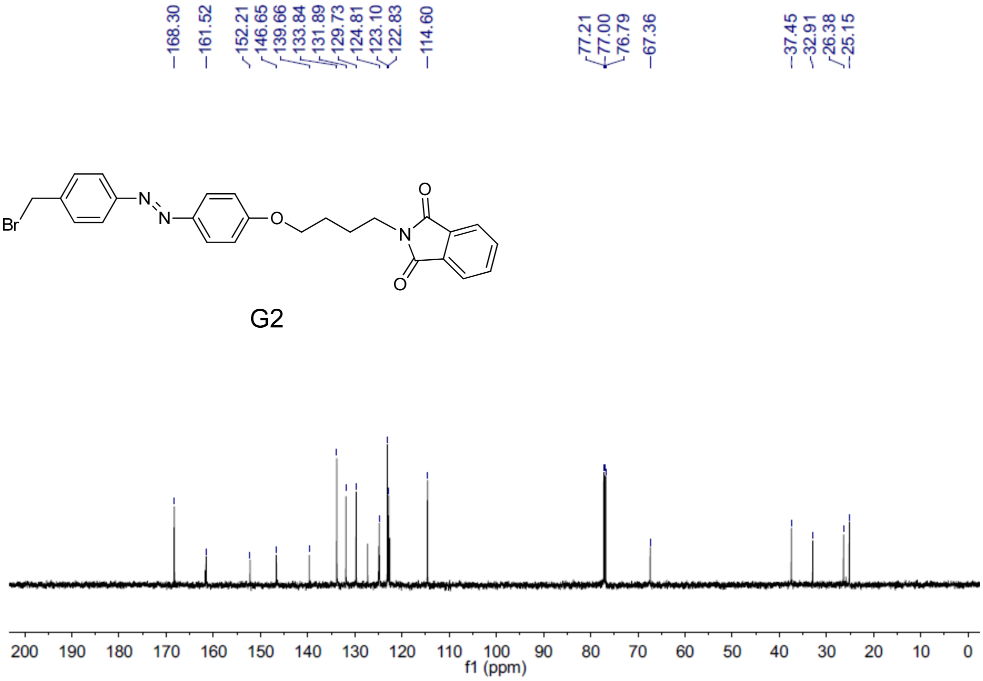

Supplementary Figure 19. ^{13}C NMR spectrum (100 MHz) of **G2** in $CDCl_3$.

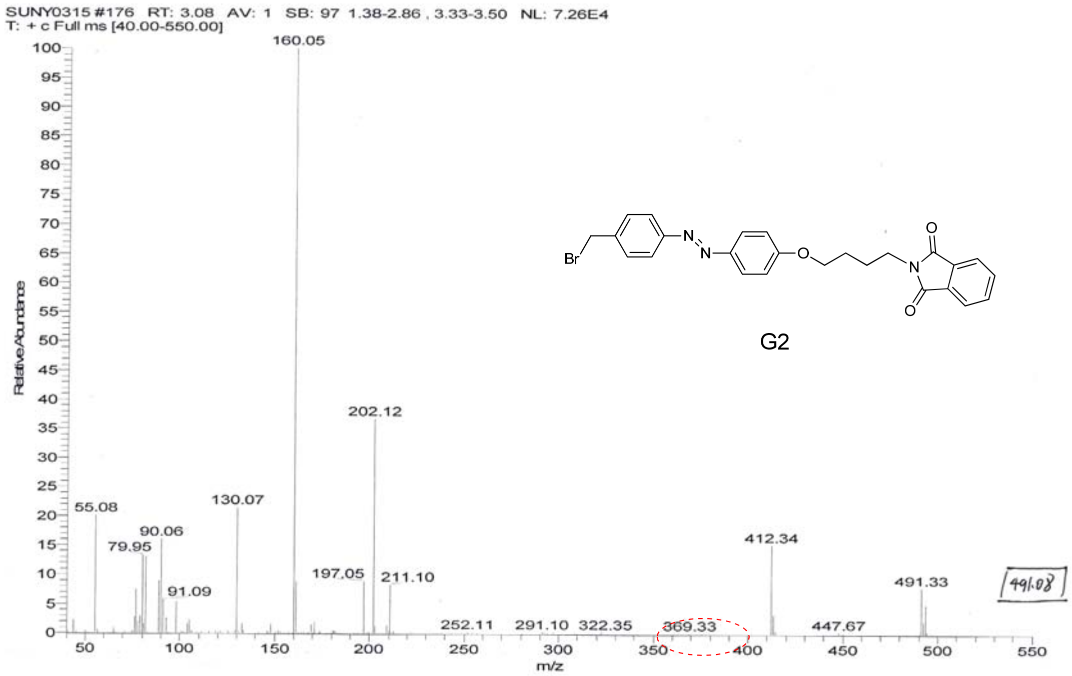

Supplementary Figure 20. Mass spectrum of compound **G2**.

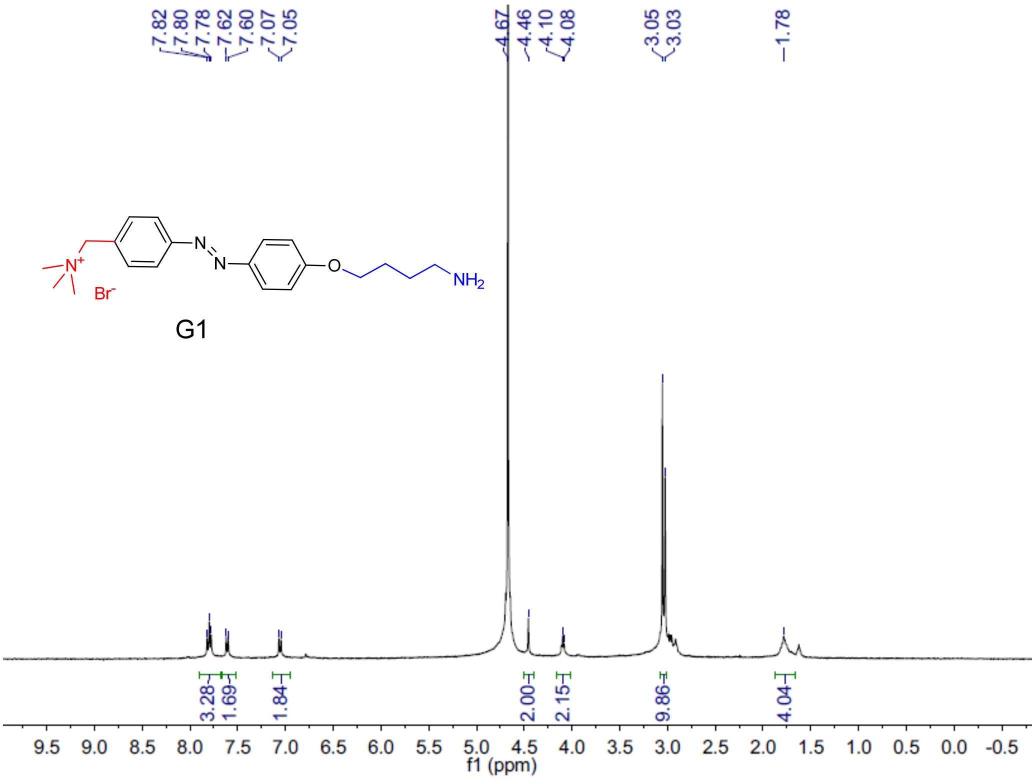

Supplementary Figure 21. ^{1}H NMR spectrum (400 MHz) of **G1** in D_2O.

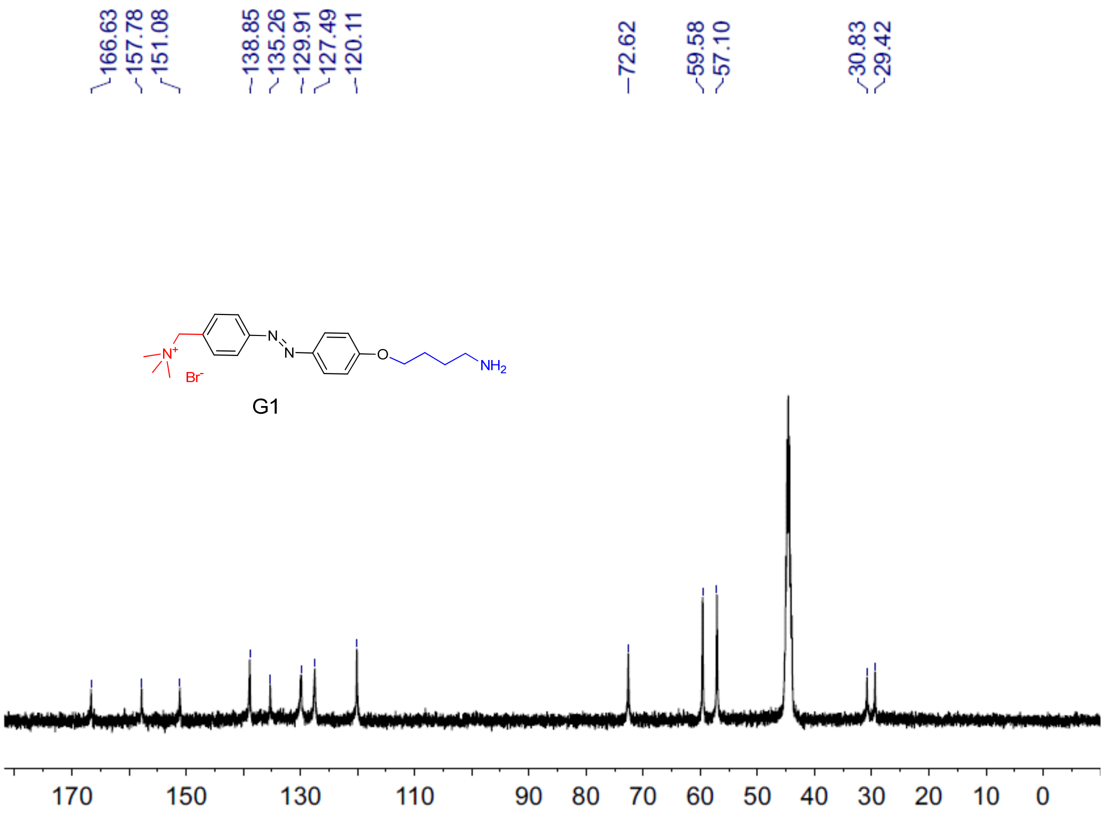

Supplementary Figure 22. ^{13}C NMR spectrum (100 MHz) of **G1** in DMSO.

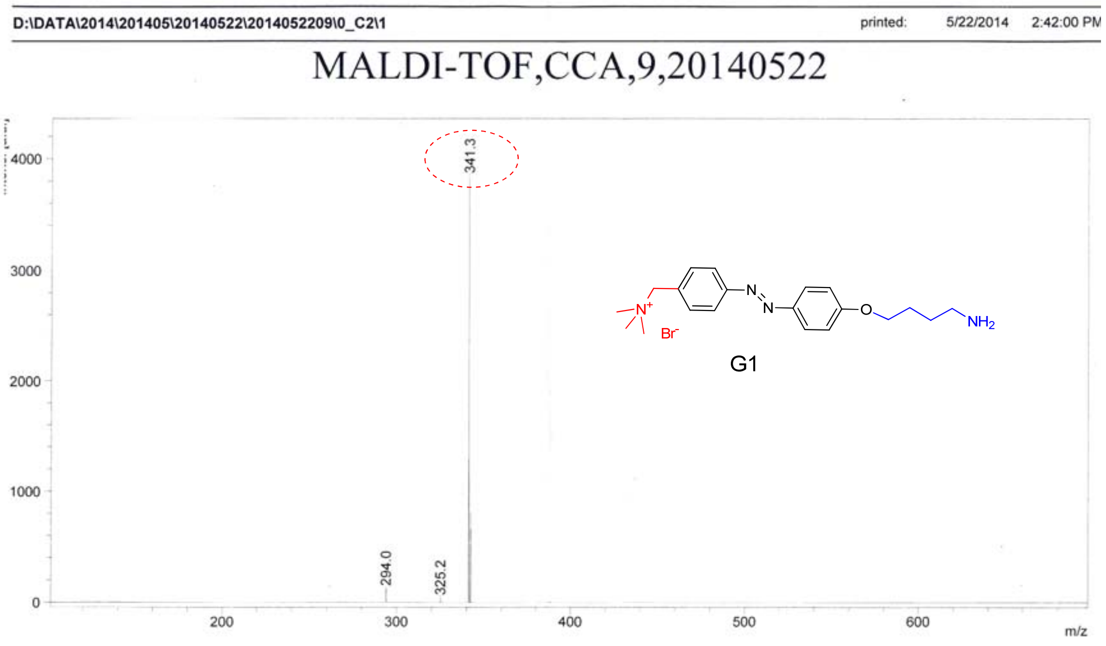

Supplementary Figure 23. Mass spectrum of compound **G1**.

Supplementary methods

Materials

Poly (ethylene terephthalate) (PET, 12 μm thick) membranes were irradiated with single heavy ion (Au) of energy 11.4 MeV/nucleon at UNILAC linear accelerator (GSI, Darmatadt, Germany). 1-Ethyl-3-(3-dimethyllaminopropyl) carbodiimide hydrochloride (EDC·HCl, ≥98.5%), N-hydroxysulfosuccinimide (NHS, ≥98.0%), sodium hydroxide (NaOH), hydrochloric acid (HCl), formic acid (HCOOH), potassium chloride (KCl) were purchased from Sinopharm Chemical Reagent Shanghai Co., Ltd. (SCRC, China). All chemical reagents were all used as received, electrolyte solution were prepared in MilliQ water (18.2 MΩ). Current-voltage curves were measured by a Keithley 6487 picoammeter (Keithley Instruments, Cleveland, OH). For UV light irradiation, a 300 W xenon lamp was used with a 365 nm filter. The intensity was measured with an optical power/energy meter (Model 842-PE). For this work, a custom-built, photoelectro-chemical cell was adopted, which can be irradiated from both sidewalls. Scanning electron microscopy (SEM) investigations were carried out on a JEOL 6390LV instrument.

Synthetic method and Characterization of P6A and AZO.

Reagents were commercially available and used as received. Solvents were either employed as purchased or dried according to procedures described in the literature. ^{1}H and ^{13}C NMR spectra were recorded on a Mercury-Plus spectrometer (400 MHz). MALDI-TOF-TOF were recorded on a Synapt G2 HDMS system (Waters,USA). Elemental analyses were performed on a Perkin-Elmer 240 C analyzer.

Synthesis of compound (G3): Compound G4 was synthesized according to the literature.[2] Phthalimide (0.184 g, 1.25 mmol) and K_2CO_3 (0.345 g, 2.5 mmol) was added to a solution of G4 (266 mg, 1.00 mmol) in dry N, N-dimethylformamide (30 mL). The reaction mixture was stirred at ambient temperature for 12h under the protection of nitrogen atmosphere. Then the DMF solvent was removed under vacuum to give buff solid. The residue was dissolved with chloroform. The organic layer was washed with H_2O. The organic layer was dried over Na_2SO_4. After the solvent was evaporated, the residue was purified by column chromatography (silica gel, hexane–dichloromethane, 1 : 1) to give G3

as yellow product (363 mg, yield: 88%). ^{1}H NMR (400 MHz, $CDCl_3$): δ 7.87 (s, 4H), 7.79 (s, 2H), 7.73 (s, 2H), 7.72 (s, 2H), 6.99 (d, J = 12.0 Hz, 2H), 4.08 (s, 2H), 3.80 (s, 2H), 2.43 (s, 2H), 1.90 (s, 4H). ^{13}C NMR (100 MHz, $CDCl_3$): δ 168.22, 161.10, 150.61, 146.74, 140.58, 133.76, 131.87, 129.52, 124.40, 123.02, 122.38, 114.48, 67.26, 37.42, 26.38, 25.14, 21.32. MALDI-TOF-MS: caclulated for $C_{25}H_{23}N_3O_3$: 413.17, found 413.31. Anal. Calcd for $C_{25}H_{23}N_3O_3$: C, 72.62; H, 5.61; N, 10.16; found: C, 72.66; H, 5.57; N, 10.17.

Synthesis of compound (G2): A mixture of G3 (413mg, 1 mmol), N-bromosuccinimide (0.25 g, 1.4 mmol) and benzoyl peroxide (10 mg, 0.042 mmol) in CCl_4 (20 mL) was heated at reflux for 12 h. The mixture was cooled to room temperature and washed with water (2x30 mL), dried (Na_2SO_4) and concentrated in vacuo. The residue was purified by column chromatography (silica gel, hexane-dichloromethane, 1 : 1) to give compound G2 (360 mg, yield: 73%). ^{1}H NMR (400 MHz, $CDCl_3$): δ 7.90 (m, J = 8.0 Hz, 6H), 7.85 (d, J = 4.0 Hz, 2H), 7.73 (d, J = 4.0 Hz, 2H), 7.00 (d, J = 12.0 Hz, 2H), 4.56 (s, 2H), 4.09 (s, 2H), 3.79 (s, 2H), 1.90 (s, 4H). ^{13}C NMR (100 MHz, $CDCl_3$): δ 168.30, 161.52, 152.21, 146.65, 139.66, 133.84, 131.89, 129.73, 124.81, 123.10, 122.83, 114.60, 67.36, 37.45, 32.91, 26.38, 25.15. MALDI-TOF-MS: Calcd for $C_{25}H_{22}N_3O_3Br$: 491.33; found 491.33. Anal. Calcd for $C_{25}H_{22}N_3O_3Br$: C, 60.98; H, 4.50; N, 8.53; found: C, 60.99; H, 4.53; N, 8.49.

Synthesis of compound (G1): A solution of G2 (493 mg, 1 mmol) in ethanol (50.0 mL) and trimethylamine (30% in ethanol, 10.0 mL) was allowed to react at 80 °C for 24 h. After that, hydrazine hydrate was added to the mixture, to futher reaction for 12 h. The solution was concentrated under reduced pressure. The residue was diluted with water (20.0 mL) and washed with dichloromethane. Then, removed water in vacuo to give a organe solid. (145 mg, 41%).^{1}H NMR (400 MHz, $CDCl_3$): δ 7.82 (m, J = 8.0 Hz, 4H), 7.62 (d, J = 8.0 Hz, 2H), 7.07 (d, J = 8.0 Hz, 2H), 4.46 (s, 2H), 4.10 (d, J = 8.0 Hz, 2H), 3.05 (s, 9H), 1.78 (s, 4H). ^{13}C NMR (100 MHz, DMSO): δ 166.63, 157.78, 151.08, 138.85, 135.26, 129.91, 127.49, 120.11, 72.62, 59.58, 57.10, 30.83, 29.42. MALDI-TOF-MS: Calcd for $C_{20}H_{29}N_4OBr$: 420.15. Found: 341.3 [M-Br$^-$]. Anal. Calcd for $C_{20}H_{29}N_4OBr$: C, 57.01; H, 6.94; N, 13.30; found: C, 56.97; H, 6.98; N, 13.30.

The interaction between AZO and P6A

To determine the stoichiometry and association constant (K_a) between P6A and AZO. ^{1}H

NMR titrations were done with solutions which had a constant concentration of AZO (4 mM) and varying concentrations of P6A. By a mole ratio plot, a 1:1 stoichiometry was obtained, which indicated that P6A and AZO formed a 1:1 complex. Using the nonlinear curve-fitting method, the association constant was obtained for each host-guest combination from the following equation : [3]

$$\Delta\delta = (\Delta\delta_{\infty}/[H]_0)\ (0.5[G]_0 + 0.5([H]_0 + 1/K_a) - (0.5\ ([G]_0^2 + (2[G]_0(1/K_a - [H]_0)) + (1/K_a + [H]_0)^2)^{0.5}))$$

Where is the chemical shift change of H_1 of AZO at $[H]_0$, is the chemical shift change of H_1 when the guest is completely complexed, $[G]_0$ is the fixed initial concentration of the guest AZO, and $[H]_0$ is the varying concentrations of host P6A.

The photocontrollable threading–dethreading behavior between P6A and AZO

To confirm photocontrollable threading–dethreading behavior, ^{1}H NMR characterization was conducted to provide evidence about the interaction of *trans*-AZO and *cis*-AZO with P6A. Compared with free *trans*-AZO (Supplementary Figure 4A), significant chemical shift changes of the signals for the protons on *trans*-AZO occurred in the presence of an equimolar amount of P6A (Supplementary Figure 4B). The peaks related to Ha, Hb, Hc, Hd shifted upfield remarkably (-0.96, -0.48, 0.56, -0.68 ppm, respectively). Moreover, these peaks became broad owing to complexation dynamics. The reason for the extensive changes of the chemical shifts is that these protons are located within the cavity of P6A and are shielded by the electron-rich cyclic structure upon forming a threaded structure between P6A and *trans*-AZO. Additionally, the protons on P6A also exhibited chemical shift changes. The peak related to H_1 shifted downfield from 6.54 to 7.12 ppm. These evidences show the formation of an inclusion complex between P6A and *trans*-AZO (The following picture).

As shown in Supplementary Figure 4E, the molar ratio of the *trans* to *cis* form of AZO changed to 50 : 50 after irradiation with UV light at 365 nm for 15 min. And the chemical shift of proton Ha* of *cis*-AZO shifted upfield from 7.01 to 5.41 ppm in the presence of equimolar P6A (Supplementary Figure 4D). The peak exhibited a broadening effect, suggesting the complexation between P6A and *cis*-AZO. Moreover, the chemical shifts of protons Hb*, Hc*, and Hd* on the benzene rings of *cis*-AZO changed slightly, indicating that the benzene ring containing protons Hc*, Hb* and Hd* of guest *cis*-AZO was outside the cavity of P6A. However, upon irradiation with light at 435 nm for 15 min, *cis*-AZO went back to *trans*-AZO, and the proton signals related to the solution of P6A and AZO went

back to the original state (Supplementary Figure 4F), suggesting that the photo-controllable *threading–dethreading* switch between P6A and AZO was achieved.

Molecular stimulation

The binding of P6A and AZO were examined by computational calculations at b3Lyp/6-31G(d) levels by using Gaussian 03.

Computational model of P6A and tran-AZO

%chk=P6A and *trans*-AZO.chk

%mem=10GB

%nprocshared=8

opt b3lyp/6-31g(d) geom=connectivity

P6A and *trans*-AZO

-11 1

Cartesian Co-ordinates (XYZ format) (a part of the data)

C	-5.07393100	-1.47097500	-0.27616800
C	-4.83342800	-0.73256800	-1.42878400
C	-4.79673300	0.65702900	-1.37487400
H	-4.50113400	1.21746800	-2.23848400
C	-5.15077200	1.37264400	-0.23537200
C	-5.57365200	0.63117100	0.87265900
C	-5.45548600	-0.75730900	0.85920000
H	-5.69467500	-1.31322400	1.74332100
C	-5.44191400	-1.10797300	-3.78659400
H	-4.78151200	-1.12034100	-4.64264500
H	-5.85699900	-0.11070900	-3.70651400
C	-6.62084100	-2.07704800	-4.10045600
C	-1.32631400	-6.99739000	-3.83913000
C	1.70055000	7.61581400	-3.31858600
C	1.86350000	-7.40027400	3.72699800
C	5.85128800	-5.31779500	-3.23707300
C	8.28165100	1.98623300	-3.39713200
C	-5.62262200	-6.05274600	3.44654600
C	-7.13374100	0.68409900	2.78018500
H	-6.66389500	0.21957800	3.63796100
H	-7.63663100	-0.10387600	2.22896400
C	-8.25971400	1.62656600	3.32411900
C	-2.69192200	7.52911500	3.96063100
O	9.53401300	2.03842700	-3.19612400

O	7.66803200	2.28640900	-4.43342800
C	6.64662900	-1.73401500	4.11483800
C	5.47113700	5.49123300	3.47617400
C	-5.20707400	2.91509300	-0.24081400
H	-5.83753700	3.20237100	0.58900900
H	-5.66082000	3.25430400	-1.16426900
C	-3.86893300	3.67143000	-0.12493600
C	-3.10606900	3.94036700	-1.25569900
C	-1.88540400	4.59621300	-1.13579500
H	-1.25629000	4.71749500	-1.99491200
C	-1.44089800	5.13487400	0.06698700
C	-2.28856700	5.01300700	1.17210300
C	-3.43916300	4.23381600	1.07534400
H	-4.04174300	4.07618300	1.94713800
C	-3.72303600	4.48739200	-3.57929600
H	-3.37724400	3.99402800	-4.47708600
H	-3.08451400	5.34581000	-3.40945900
C	2.00818100	6.09077700	-3.22777500
H	3.06819800	5.99073700	-3.02744600
H	1.81830500	5.66524000	-4.20368000
O	2.48883900	-7.83344300	4.74313100
O	1.09831700	-8.02512000	2.97539100
O	-5.49778800	-6.87423700	4.40466300
O	-6.67081000	-5.68411300	2.89332200
O	-8.97171700	1.02603800	4.18446000
C	4.02743400	4.90894300	3.40934400
H	3.80539300	4.48968400	4.38111000
H	3.35399500	5.74241800	3.24940600
O	-8.40550400	2.77780200	2.88491000
O	7.14664900	-1.36984800	5.22127300
O	6.99534100	-2.67953600	3.38880100
O	5.59723000	6.27713200	4.46238900
C	5.05212100	3.07451300	0.00856500
H	5.53384700	3.36316800	-0.91519700
H	5.61294100	3.48388400	0.84055800
C	5.08830300	1.53314400	0.05791400
C	5.52304400	0.77698000	-1.02781400
C	5.46936900	-0.61585800	-0.97288700
H	5.72564200	-1.17312400	-1.85184000
C	5.12736800	-1.30788800	0.18144600
C	4.86978800	-0.54186400	1.32076000
C	4.77875800	0.84007000	1.23003100
H	4.48437100	1.41541200	2.08483400
C	7.55112500	1.44703100	-2.12323200

H	7.94303600	0.45308100	-1.93783600
H	7.86774700	2.06851100	-1.29328300
O	6.31804100	5.18600200	2.62113700
O	-3.60037100	7.79350900	4.80452500
O	-1.69138600	8.20687200	3.68046200
C	5.47099000	-0.79988600	3.69741600
H	4.78257700	-0.75731200	4.53027300
H	5.88225900	0.19294000	3.56042900
C	5.17920100	-2.84474600	0.26876700
H	5.78236200	-3.22575800	-0.54594300
H	5.65883300	-3.07290100	1.21285600
C	3.83232200	-3.59076500	0.19536000
C	3.32782200	-4.03237800	-1.02539700
C	2.09646000	-4.67955100	-1.08045600
H	1.66052800	-4.91795500	-2.02932300
C	1.39521100	-5.05061600	0.06428100
C	1.98119200	-4.74461000	1.29097900
C	3.13340400	-3.96427600	1.34021600
H	3.51704400	-3.63934500	2.28603600
C	4.35532100	-4.90399800	-3.08804800
H	4.00336600	-4.61545800	-4.06986800
H	3.80593200	-5.79162400	-2.79528500
C	2.19850700	-5.90114800	3.45624700

Computational model of P6A and *cis*-AZO

%chk=P6A and *cis*-AZO.chk

%mem=10GB

%nprocshared=8

opt b3lyp/6-31g(d) geom=connectivity

P6A and *cis*-AZO

-11 1

Cartesian Co-ordinates (XYZ format) (a part of the data)

H	-2.96565100	0.34979100	-0.45194700
N	3.92259900	-0.18652200	-0.23829200
N	3.48208100	0.97272900	-0.34184400
C	2.09184400	1.15646800	-0.20318500
C	1.19062700	0.13016600	0.03336300
C	-0.15519400	0.39640800	0.15095800
C	-0.62004200	1.70136500	0.03127700

C	0.28507200	2.73311500	-0.20462900
C	1.62358500	2.46003000	-0.32030600
O	-1.92308700	2.05761100	0.12857000
C	-2.98305000	1.09011300	0.33780100
H	9.73241400	-1.99001600	-1.21849800
H	9.97851000	-0.27198200	-1.50978100
H	8.18214600	1.34953000	-1.04469200
H	5.16944900	-2.39906000	-0.09202400
H	5.77192300	1.72575600	-0.77867600
H	-0.09647200	3.72734300	-0.29382500
H	-0.83438900	-0.40854600	0.33194800
H	2.32817000	3.24430200	-0.50489700
H	1.55524600	-0.87084500	0.12098500
C	5.33206100	-0.31288600	-0.39391200
C	-4.28700800	1.87764900	0.31092300
C	6.18581300	0.74739200	-0.66664900
C	7.53829400	0.52537900	-0.80404400
C	5.84678000	-1.59223500	-0.27738000
C	8.06590800	-0.75498900	-0.65931900
C	9.54036100	-0.99690500	-0.83937200
H	7.57920800	-2.81725600	-0.35037600
N	10.36830200	-0.89406100	0.46727400
C	11.81955800	-1.15832800	0.12551800
C	9.90361700	-1.91971400	1.47766300
C	10.24645800	0.48756000	1.07168700
H	12.40701900	-1.08981300	1.02819500
C	7.20228700	-1.81480100	-0.41483300
H	12.14986800	-0.41943100	-0.58824900
H	11.90379200	-2.14776300	-0.29693500
H	10.52002200	-1.83364400	2.35941400
H	10.85662600	0.52581000	1.96111400
H	10.00990100	-2.90383700	1.04731200
H	8.87242400	-1.72595900	1.71802200
H	9.21367700	0.66906900	1.31478000
H	10.59555300	1.21298800	0.35276700
H	-4.36282400	2.37060300	-0.65086300
H	-4.23965700	2.64442100	1.07519300
C	-6.82195500	1.72970600	0.40110400
H	-5.45106800	0.50432600	1.51992700
H	-5.50988800	0.16788700	-0.20547200
N	-7.98419100	0.86025800	0.60117500
H	-6.87825900	2.16638800	-0.58708000
H	-6.87204600	2.52292100	1.13553900
H	-8.55874700	1.01063900	1.39763600

C	-8.34556600	-0.02278100	-0.35894900
C	-9.54355600	-0.87816300	-0.09557200
O	-7.72327600	-0.10815100	-1.41023100
C	-10.10837500	-1.05709500	1.15776500
C	-11.21414100	-1.87361500	1.31112200
C	-11.75865000	-2.51552700	0.21230600
C	-11.18846500	-2.35007900	-1.03862900
C	-10.08050700	-1.53995100	-1.18860200
H	-9.68498000	-0.59063700	2.02457800
H	-11.64458800	-2.01228700	2.28253900
H	-12.61768400	-3.14513500	0.33227400
H	-11.60360700	-2.85165600	-1.88948300
H	-9.61479200	-1.40532500	-2.14223500

Fabrication of single conical nanochannel

The single conical nanochannel was prepared in a PET polymer film using the well-known ion track etching technique. Before etching process, each side of the PET membranes were exposed in UV light (365 nm) for 1 h. In order to obtain the conical nanochannel, etching was performed only from one side, the other side of the cell contains a solution that is able to neutralize the etchant as soon as the pore opens, thus slowing down the further etching process. The PET membrane was embedded between the two chambers of a conductivity cell at 30 °C, one chamber was filled with etching solution (9 M NaOH), the other chamber was filled with stopping solution (1 M KCl + 1 M HCOOH). Then a voltage of 1 V was applied across the membrane. The etching process was stopped at a desired current value corresponding to a certain tip diameter. The membrane was immerged in MilliQ water (18.2 MΩ) to remove residual salts.

SEM characterization of nanochannel

The diameter of the base was estimated from the multitrack membrane by field-emission scanning electron microscopy (FESEM) which was etched under the same conditions as the single-channel sample. The diameter of large opening of conical nanochannel which was called base (D) was determined by scanning electron microscopy (SEM). The diameter of the small opening which was called tip (d_{tip}) was estimated by the following relation:

$$d_{tip} = \frac{4LI}{\pi k(c)UD}$$

L is the length of the pore, which could be approximated to the thickness of the

membrane after chemical etching; I is the measured ion current; U is the applied voltage; d_{tip} and D is the tip diameter and the base diameter respectively; k(c) is the specific conductivity of the electrolyte. For 1 M KCl solution at 25 °C, k(c) is 0.11173 Ω^{-1} cm^{-1}. In this work, the base diameter is about 600 nm and the tip diameter is about 20 nm, which was further confirmed by SEM.

Ion currents measurement.

Ion currents were measured by a Keithley 6487 picoammeter (Keithley Instruments, Cleveland, OH). Ag/AgCl electrodes were used to apply a transmembrane potential across the film. The film was mounted between the two halves of the conductance cell. Both halves of the cell were filled with a 0.1 M KCl solution prepared. In order to record the *I–V* curves, a scanning triangle voltage signal from –2V to +2V with a 40s period was selected. Each test was repeated 5 times to obtain the average current value at different voltage. Specifically, before exposure to UV radiation, the transmembrane currents were obtained in a 0.1 m KCl solution under a scanning triangle voltage signal from –2V to +2V. Upon irradiation with UV light, the functional nanochannel was fixed in the halves of the cell. This process was supported further by applying a potential of +5 V on the side containing a 0.1 m KCl solution for 1h. Then the PET film was immersed in methanol for 5 h. After that, the functionalized channels were further washed several times with distilled water. To measure the resulting ion current flowing through the nanochannel, a scanning voltage between -2 to +2 V on the two sides was applied.

The modification of light-controlled nanochannel

As a result of chemical etching, carboxyl groups are generated on the nanochannel surface. These can be activated with EDC/NHS, forming an amine-reactive ester intermediate. Then these reactive esters were further condensed with AZO through the formation of covalent bonds. In this paper NHS ester was formed by soaking PET film in an aqueous solution of 30 mg EDC and 6 mg NHS for 1 hour. After that washing this film with distilled water and treated it with 1 mM AZO solution overnight. Then, the P6A were attached to the AZO-channel by self-assembling. Finally, the modified-film was washed three times with distilled water.

Contact angles measurement

Contact angles were measured using an OCA20 (DataPhysics, Germany) contact angle system at ambient temperature and saturated humidity. The original PET membrane for contact angle measurement was treated with NaOH (9 M) at 38 °C for 50 min. And then the sample was removed from the etching solution and treated with the stopping solution (1 M HCOOH) for 20 min. After that, the sample was treated with distilled water overnight. The modification process on the PET film is same to the modification process in the inner wall of the nanochannel. Before the contact angle test, the sample was blown dry with N_2. In each measurement, an about 1μL droplet of water was dispensed onto the surface of PET membrane. The average contact angel value was obtained at five different positions of the same membrane. As shown in Supplementary Figure 10, the change of the wettability of the surface means the change of the chemical composition, to some extent, which indicated the successful modification of the AZO and P6A.

XPS experiment

X-ray photoelectron spectra (XPS) data were obtained with an ESCALab220i-XL electron spectrometer from VG Scientific using 300 W Al K_α radiation. In this work, all peaks were referenced to C1s (CHx) at 284.8eV in the deconvoluted high resolution C1s spectra. The chemical functionalization of carboxyl ($–COO^-$) groups generated on the channel surface during the track-etching process were modified by the following procedure: for the activation of carboxyl groups into NHS-ester, the single-channel contained PET film was exposed to an aqueous solution of 15 mg EDC and 3 mg NHS for 1 h at room temperature. After washing with distilled water, the samples were further treated with 5 mM AZO for an overnight time period. Then, the PET film was immersed in 10^{-3} M P6A solution for 5 h. After that, functionalized channels were washed several times with distilled water and fabricated successfully.

Laser scanning confocal microscopy experiment

To further confirm switching between threading and dethreading states by alternating visible and ultraviolet light in the nanochannel, the fluorescence of the nanochannel is observed in site by laser scanning confocal microscopy (Supplementary Figure 12). We used the P6A fluorescent derivative (P6A-RhB), which was synthesized by linking the amino group to the rhodamine B amine ($RhB-NH_2$). A host–guest complex was then

formed on the AZO-modified porous PET membrane by the interaction between AZO and P6A-RhB. As shown in the following picture, when the P6A-RhB successfully assembled on the AZO-immobilized nanochannel, the nanochannel exhibited a strong fluorescence signal. The fluorescence thickness was ca. 13.0±0.5 μm, which agreed with the actual thickness of the PET membrane. Subsequently, the functional nanochannel was further irradiated under the UV light. And we handled the functional nanochannel accordance with the above experimental steps (supporting information in **9** Ion currents measurement). The fluorescent in the nanochannel weakened, which is likely to provide further evidence of the release of P6A.

Molecules transport of the ATP

ATP served as the cargo. A nanochannel-containing membrane (still mounted in the etching cell) was exposed to the electrolyte solution on one side (permeate side) and electrolyte solution to which 100 mM ATP had been added on the opposite side (feed side). This was accomplished by periodically measuring the UV absorbance of the ATP in the permeate solution and making plots of moles of ATP transport vs time. We may caculate the Flux. For ^{31}P NMR experiments, we directly investigate the permeating side after 90 min before and after UV light irradiation.

Supplementary references

1. Yao, Y.; Li, J. Y.; Dai, J.; Chi, X. D.; Xue, M. *RSC Adv*., **4**, 9039–9043 (2014).

2. Ito, M.; Wei, T. X.; Chen, P. L.; Akiyama, H.; Matsumoto, M.; Tamadab, K.; Yamamoto, Y. *J. Mater. Chem.,* **15**, 478–483 (2005).

3. Li, C.; Zhao, L.; Li, J.; Ding, X.; Chen, S.; Zhang, Q.; Yu, Y.; Jia, X. *Chem. Commun*., **46**, 9016–9018 (2010).
